# Update of the diagnostic criteria of brain death: application and training of physicians

**DOI:** 10.5935/0103-507X.20190055

**Published:** 2019

**Authors:** José Antonio Chehuen Neto, Renato Erothildes Ferreira, Igor Malheiros Assad, Ivy Alves Santos, João Luís Carvalho Tricote dos Santos, Luíza Campos de Paula, Sávio Dornelas Breder

**Affiliations:** 1 Faculdade de Medicina, Universidade Federal de Juiz de Fora - Juiz de Fora (MG), Brasil.

**Keywords:** Brain death/diagnosis, Brain death/legislation & jurisprudence, Tissue and organ procurement, Physician's role, Ethics, medical

## Abstract

**Objective:**

To evaluate the medical knowledge regarding the application of the diagnostic criteria for brain death and to correlate it with training parameters for this diagnosis according to Federal Council of Medicine resolution 2,173 of 2017.

**Method:**

We interviewed 174 physicians with experience with comatose patients. A structured questionnaire adapted from previous studies was used. The associations of the variables were tested using the chi-square test for independence. A multivariate logistic model was fitted for associations with p values ≤ 0.20.

**Results:**

Among the interviewees, 40% had been working for more than 1 year in intensive care, and 23% had initiated ten or more brain death protocols complying with the new resolution. Forty-five percent of the interviewees reported having difficulty following the criteria, 94% acknowledged the need for complementary tests for diagnosis, and 8% of them reported the existence of incorrect tests. The difficulty with these criteria decreased with an increase in the number of years of medical training (OR = 0.487; p = 0.045; 95%CI 0.241 - 0.983) and with a higher number of initiated brain death protocols (OR = 0.223; p = 0.0001; 95%CI 0.117 - 0.424).

**Conclusions:**

Difficulties in the application of brain death criteria were identified by a significant portion of the sample. However, among other factors, more years of training and a greater number of initiated brain death protocols were associated with greater ease in the application of brain death criteria according to the guidelines provided in Resolution 2,173 of the Federal Council of Medicine.

## INTRODUCTION

Brain death (BD) can be defined as the irreversible cessation of brain cortical and brainstem functions^(1)^ as well as the inability to stay alive without artificial support.^(2,3)^ Although this concept is widely disseminated, there are doubts among health professionals about the diagnostic criteria.^(2-4)^

A new resolution from the Federal Council of Medicine (*Conselho Federal de Medicina* - CFM), resolution 2,173 from 2017, updated the criteria for the diagnosis of BD. According to the previous standards, which were in force from 1997 to 2017, BD should be confirmed by two physicians, one of whom must be a neurologist, while the second was not required to have any specific qualification.^(5)^ Under the new resolution, the two physicians must be duly trained, and one of them must be from one of the following specialties: adult or pediatric intensive care, adult or pediatric neurology, neurosurgery or emergency medicine. The other physician must have at least 1 year of experience in the care of comatose patients, having participated in or performed at least ten diagnoses of BD or participated in specific training for this purpose in a program that meets the standards determined by the CFM.^(6)^ The new resolution also discusses, in more detail than the previous one, the clinical prerequisites for the diagnosis of BD and intends to guide its application, aiming to ensure greater confidence in the diagnosis of BD.^(6)^

The new resolution also lists and standardizes the diagnostic criteria for BD. The minimum interval between clinical evaluations, which was previously 6 hours for individuals older than 2 years, is now 1 hour. The required complementary test can be performed at any time during the diagnosis of BD as long as the specific clinical criteria for its correct diagnosis are met and not only at the end of the BD protocol, thus reducing unnecessary expenses.^(6)^

It should also be noted that the registration of cases of BD is the most important method for evaluating the rate of potential organ donors, according to the *Associação Brasileira de Transplante de Órgãos* (ABTO).^(7)^ Accurate and timely diagnosis of BD, coupled with an adequate explanation of the patient's condition to his/her relatives by a health professional, can increase the number of transplants in the country and avoid the costs of unnecessary interventions, avoid prolonging family and patient suffering, and increase the supply of transplant organs.^(8,9)^

This study of the new guidelines for BD diagnosis is unprecedented in the region and infrequent in the country.

This study aimed to evaluate medical knowledge in the application of the diagnostic criteria of BD and to correlate it with training parameters for this purpose according to CFM resolution 2,173 from 2017.

## METHODS

The study design was cross-sectional, descriptive and quantitative^(10)^ and involved the analysis of field collected data. This was an original applied study conducted in the city of Juiz de Fora, state of Minas Gerais (MG). Brazil. The study area was delimited based on the city's geographic area with the highest concentration of intensive, urgency and emergency medical facilities, being the centralization of medical assistance a characteristic of the city. Thus, a plural and representative sample of the entire city was characterized for this study.

The sample size calculation considered a prevalence of knowledge about the concept and diagnosis of BD of 85%,^(2)^ a maximum sampling error of 5.0% with a 95% confidence interval, a maximum alpha variance of 0.05, and a power of 70%. Under these conditions, for finite populations, a sample size of 195 interviewed physicians was calculated. For the selection of the participants, a simple random sample was drawn. Data collection was performed between April 1, 2017, and December 1, 2017. The physicians most likely to be involved in the initiation of the BD protocol, i.e., those who worked in the adult or pediatric intensive care unit (ICU), the coronary unit and the urgent care and emergency sectors were approached.

The instrument used for data collection was a structured questionnaire (Appendix 1) adapted from previous studies.^(2,3,11,12)^ The questionnaire was applied through a face-to-face interview standardized by a trained researcher and divided into two sections. The first section contained 7 questions regarding the identification of the professional profile of the physicians participating in the study, and the second section consisted of 11 questions related to knowledge of BD, diagnostic criteria and protocol application.

The criteria for inclusion in the sample were having an undergraduate degree in medicine and practicing at a hospital or another setting with an ICU or emergency and urgent care at the time of the interview, in accordance with the criteria defined by law 3,268/57^(13)^ for professional qualification. The exclusion criteria were interruption of the questionnaire for any reason or missing data.

The investigated variables were divided into three groups: continuous quantitative, dichotomous qualitative and ordinal variables. Descriptive and exploratory statistical analyses of the data were performed using absolute frequencies (n) and relative frequencies (%). For the comparative analysis of the dichotomous qualitative variables, 2 × 2 contingency tables were generated containing the absolute (n) and relative (%) frequencies. To test the association between the variables, the chi-square test of independence (without correction) was performed. The significance level adopted for this test was p-value ≤ 0.05 for a 95% confidence interval (95%CI). For the prevalence data, the term "odds" was adopted. For the analysis of data with binary outcomes, univariate and multivariate binary logistic regression was used to estimate the odds. In this technique, the dependent variable (outcome) is a random dichotomous variable that takes the value (1) if the event of interest occurs or (0) of it does not.

The prevalence of difficulty with the BD criteria is presented as a percentage and was also adjusted within each category of the variables of interest, followed by the univariate odds ratio (OR) and its 95% CI. Finally, a multivariate logistic model was fitted considering associations with p values ≥ 0.20 for the final model. The final multivariate model that best explained the study objective was selected based on the goodness of fit, as given by the Akaike information criterion (AIC). The model with the best fit had the lowest AIC values (44 - 45). The level of significance was alpha ≤ 0.05 for a 95% CI. Analyses were performed using STATA 15 (Data Analysis and Statistical Software, College Station, Texas, USA).

The study was approved by the Human Research Ethics Committee of the *Universidade Federal de Juiz de Fora* under number 1945938.

## RESULTS

A total of 196 physicians were approached, and 22 refused to participate (11.2%). Thus, 174 physicians effectively answered the questionnaire. Table 1 shows the profile of the study participants.

**Table 1:** Knowledge about brain death

Demographic characteristics of the sample	Prevalence 174 (100%)	Has difficulty with BD criteria 78 (44.83)	No difficulty with MD criteria 96 (55.17)	OR (95% CI)	p value
Sex					
Female	97 (55.25)	48 (61.54)	30 (38.46)	Reference	0.166
Male	77 (44.75)	49 (51.04)	47 (48.56)	0.65 (0.35 - 1.19)	
Age, years					
24 - 30	51 (29.31)	32 (41.03)	19 (19.79)	Reference	0.005
31 - 41	72 (41.38)	30 (38.46)	42 (43.75)	0.42 (0.20 - 0.88)	
42 - 66	51 (29.31)	16 (20.51)	35 (36.46)	0.27 (0.11 - 0.61)	
Training, years					
0 - 5	46 (26.44)	29 (37.18)	17 (17.71)	Reference	0.016
6 - 10	53 (30.46)	24 (30.77)	29 (30.21)	0.48 (0.21 - 1.08)	
11 - 18	31 (17.82)	11 (14.10)	20 (20.83)	0.12 (0.45 - 0.83)	
> 19	44 (25.29)	14 (17.95)	30 (31.28)	0.27 (0.11 - 0.65)	
Medical specialty					
Clinical	123 (70.69)	52 (66.67)	71 (73.96)	Reference	0.293
Surgical	51 (29.31)	26 (33.33)	25 (26.04)	1.42 (0.73 - 2.73)	
**Questions related to knowledge about BD**					
Prior awareness of this study					
Yes	9 (5.17)	4 (5.13)	5 (5.21)	Reference	0.981
No	165 (94.83)	74 (94.87)	91 (94.79)	1.01 (0.26 - 3.92)	
Worked predominantly in the ICU for more than 1 year					
Yes	69 (39.66)	23 (29.49)	46 (47.92)	Reference	0.013
No	105 (60.34)	55 (70.51)	50 (52.08)	2.2 (1.17 - 4.13)	
What is your main or most important activity?					
On duty	92 (52.87)	33 (42.31)	59 (61.46)	Reference	0.003
On day shift	31 (17.82)	12 (15.38)	19 (19.79)	1.12 (0.48 - 2.610	
Resident/teacher	51 (29.31)	33 (42.31)	18 (18.75)	3.27 (1.60 - 6.70)	
How many times have you initiated the BD protocol?					
0 - 4	109 (62.64)	70 (89.74)	39 (40.63)	Reference	0.0001
5 - 9	25 (14.37)	4 (5.13)	21 (21.82)	0.10 (0.03 - 0.33)	
10 or more	40 (22.99)	4 (5.13)	36 (37.50)	0.06 (0.02 - 0.18)	
How do you rate your confidence in explaining to the patient’s family what BD is?					
Little or none	11 (6.32)	9 (11.54)	2 (2.08)	Reference	0.0001
Fair	52 (29.89)	35 (44.87)	17 (17.71)	0.45 (0.08 - 2.35)	
Good	38 (21.84)	12 (15.38)	26 (27.08)	0.10 (0.01 - 0.54)	
I feel confident	73 (41.95)	22 (28.21)	51 (53.13)	0.09 (0.01 - 0.48)	
Is the hospital structure adequate for establishing the diagnosis of BD?					
Yes	157 (90.23)	71 (91.03)	86 (89.58)	Reference	0.750
No	17 (9.677)	7 (9.97)	10 (10.42)	0.84 (0.30 - 2.34)	
Do you think you received sufficient information about this topic during your medical program?					
Yes	18 (10.34)	12 (15.38)	6 (6.25)	Reference	0.048
No	156 (89.66)	66 (84.62)	90 (93.75)	0.36 (0.13 - 1.02)	
Would you provide a declaration of BD based only on clinical examination?					
Yes	11 (6.32)	3 (3.85)	8 (8.33)	Reference	0.226
No	163 (93.68)	75 (96.15)	88 (91.67)	2.27 (0.58 - 8.87)	
What brain functions must be absent for a person to be declared brain dead?					
Incorrect answer	51 (29.31)	29 (35.90)	23 (23.96)	Reference	0.085
Correct answer	123 (70.69)	50 (64.10)	73 (76.04)	0.56 (0.29 - 1.08)	
Is there a legal need for complementary tests to establish the diagnosis of BD?					
No	11 (6.32)	8 (10.26)	3 (3.13)	Reference	0.053
Yes	163 (93.68)	70 (89.74)	93 (96.88)	0.28 (0.07 - 1.10)	

BD - brain death; OR - odds ratios; 95%CI - 95% confidence interval; ICU - intensive care unit. The results are expressed as n (%).

Considering the medical training for diagnosis of BD defined in the new CFM resolution, 69 participants (40%) had worked predominantly in the ICU for more than 1 year, and 40 (23%) had already initiated the BD protocol ten or more times.

Approximately 123 (71%) correctly defined BD as the irreversible loss of all cortical and brainstem function. A total of 42% of the professionals considered themselves to have the highest level of confidence to explain this concept to the patient's family. The level of confidence was not significantly different between those who correctly defined BD and those who did not. It is noteworthy that among the 51 participants who incorrectly defined BD, 16 (31%) considered themselves to be at the maximum level of confidence.

A total of 78 professionals (45%) reported difficulties in following the BD criteria. When comparing clinical professionals with surgical professionals, the latter reported a greater limitation, with 51% (26/51) reporting difficulty (OR = 1.42; p = 0.293). When asked about the teaching of BD during their medical program, 156 (90%) said they did not receive sufficient information on the topic during their training.

Most patients (94%) recognized the need to use complementary tests in the diagnosis of BD, and of these, 42% correctly identified all possible diagnostic tests (arteriography, electroencephalogram and transcranial Doppler). Among those who recognized this need, almost all (162/163) reported at least one of the three correct tests as appropriate for BD diagnosis. Nevertheless, 13 professionals (8%) mistakenly indicated the use of cerebrospinal fluid examination and/or simple brain computed tomography (CT) scan as options for complementary diagnostic tests.

Data on the interviewees' approaches when faced with clinical situations of BD are shown in table 2.

**Table 2:** Clinical cases presented to the interviewees

Question 1	A 5-year-old girl is found at the bottom of a swimming pool. She initially presents with apnea and no pulse. She undergoes exhaustive resuscitation measures. After 1 week in the ICU, she does not present corneal, cough or gag reflexes. She does not respond to pain stimuli. There is no nystagmus in response to caloric tests. Apnea test is positive. The results do not change in the next 2 days. What is the correct approach?	170 (97.7)	4 (2.3)
Correct answer	Request a cerebral blood flow evaluation		
Question 2	An adult patient has a first clinical examination compatible with brain death at 12:00 pm on August 10. The second clinical examination is performed at 6:00 pm* on the same day and remains unchanged. The patient is kept on vital support until suffering cardiac arrest at 8:00 pm on August 11. What is the time of death that should appear on the death certificate?	124 (71.2)	50 (28.7)
Correct answer	Time of cardiac arrest		

ICU - intensive care unit.

* It is noteworthy that according to the new resolution of the Federal Council of Medicine, resolution 2,173 from 2017, the interval between the first and the second clinical examination may be 1 hour for the age group in question and not more than 6 hours. At the time of data collection, however, the old resolution of the Federal Council of Medicine, 1,480 from 1997, was still in place. Therefore, the clinical case remained as presented above. The change to the standards does not alter the correct answer to the question. The results are expressed as n (%).

Most of the professionals (98%) correctly chose to request a cerebral blood flow evaluation for a patient with two clinical evaluations compatible with BD, as shown in table 2. In another case, the physicians were evaluated regarding the determination of the time of death in a patient with a clinical diagnosis of BD and subsequent progression to cardiac arrest without ending the protocol due to the absence of a complementary test. In this scenario, 124 (71%) correctly pointed out that the time of death would be the one in which cardiac arrest occurred. In a third scenario, according to the attached questionnaire, the physicians were asked to determine the time of death in a patient described as an organ donor. The description of the clinical case, however, characterized the patient as an organ donor before the BD protocol was completed. It was determined that the aforementioned conceptual limitation of the scenario presented to the interviewees precluded a statistical analysis of the results.

The report of difficulty in following the BD criteria by the interviewees had a significant correlation (p < 0.05) with the variables shown in table 3. A decrease in difficulty was observed with increasing years of medical training up to 18 years of training (OR = 0.487; p = 0.045; 95%CI 0.241 - 0.983). Moreover, the greater the number of BD protocols initiated, the greater the ease the participants reported in following the criteria (OR = 0.223; p = 0.0001; 95%CI 0.117 - 0.424). The significant difference regarding the so-called protection factor among the interviewed physicians who had initiated the BD protocol five to nine times (OR = 0.1; 95%CI 0.03 - 0.33) and those who had done so more than ten times (OR = 0.06; 95%CI 0.02 - 0.18) was 0.04.

**Table 3:** Multivariate model. Factors associated with difficulty following the brain death criteria

Multivariate adjustment	OR	p value	95%CI
Age	1.083	0.042	1.002 - 1.171
Years of training	0.487	0.045	0.241 - 0.983
Confidence to explain BD	0.586	0.012	0.688 - 0.889
Initiation of BD protocol	0.223	0.0001	0.117 - 0.424
Need for complementary tests for BD diagnosis	0.643	0.016	0.449 - 0.922

OR - odds ratios; 95%CI - 95% confidence interval; BD - brain death. Adjusted for: "Did you have prior knowledge of this study?", "Worked predominantly in the ICU", "Would you provide a declaration of brain death based only on clinical examination?" and "What brain functions must be absent for a person to be declared brain dead?"

## DISCUSSION

The new CFM resolution 2,173 from 2017 on BD has fostered discussions on the topic within the medical community. In this context, the present study presents useful data for a better understanding of the current situation.

The difficulty in following the diagnostic criteria of BD reported by 45% of the sample was associated with a high percentage (90%) of professionals who indicated that the information they received on the topic during their medical program was insufficient. This association reinforces the need for better medical training, especially given the changes to the current standards for BD diagnosis. In addition, a significant proportion of the physicians considered themselves to have a low level of confidence to explain the concept of BD to family members; this presumably has a negative impact on the population's desire to donate organs, a phenomenon that a previous national study^(14)^ indicates is primarily related to a low level of education about the BD diagnosis and little trust in the organ donation process.

When asked to define the concept of BD, only 71% of the professionals were correct. This percentage is lower than those observed in other studies in the Brazilian literature, such as in Teresina (Piauí)^(2)^ and Porto Alegre (Rio Grande do Sul),^(3)^ which report success rates of approximately 85% and 83%, respectively. On the other hand, and in line with other studies conducted in Brazil,^(2,3,15)^ the need for complementary tests for the diagnosis of BD was widely known by the interviewed professionals, with approximately 94% recognizing this need.

At the end of the questionnaire used in this study, the physicians were presented with specific clinical cases. First, they were asked how they would approach a case of a patient with clinical criteria indicative of BD in two different evaluations. The accuracy rate was close to 98%, higher than the 86% rate reported for a similar clinical case in the Teresina study.^(2)^ It is possible that the high percentage of correct responses was mainly because this issue is widely disseminated and because of the implausibility of the other options presented, which implied ethical violations.

In a second case, the physicians were asked about the time of death of patients with progression to cardiac arrest before the end of the BD protocol due to the absence of a complementary test. For this case, only 71% of the professionals identified the correct time, which corresponded to the time of cardiac arrest. Even so, the accuracy rate was higher than that of Teresina,^(2)^ in which only less than two-thirds of the sample (64.5%) responded correctly. Finally, a third clinical scenario aimed to evaluate whether the physicians would correctly identify the ending time of the protocol as the time of death in a scenario of end of the BD protocol. However, because the patient was characterized as an organ donor before the completion of the BD protocol, the proposed scenario had a conceptual flaw that may have compromised the study participants' understanding of the question and invalidated its statistical analysis.

The importance of coordinated actions for better management of potential organ donors, including the use of specific protocols, was corroborated by a previous study in Joinville.^(16)^ For example, only 155 BD notifications (29.4pmp/year)^(17)^ were made between January and March 2018 in the state of Minas Gerais. Thus, any difficulty that physicians have in following the BD protocol, as was observed in this study, could be a reason for the low rate of donors in the region.

Regarding the criterion defined in CFM resolution 2,173 from 2017 of the previous initiation of more than ten protocols, it was observed that those who had initiated the protocol between five and nine times had similar ease of following the BD criteria as those who had initiated it ten or more times. Thus, perhaps the number of protocols required for a physician to be qualified is lower than stipulated, although additional studies are important to confirm this hypothesis at the national level.

It is noteworthy that the questionnaire was administered to physicians on occasions when they were not facing a clinical situation of a patient with BD. The approach of each respondent to a real case at a specific time and place and under specific care circumstances may vary from the approaches reported here. Factors related to the reasons for not initiating the BD protocol in the cases indicated according to each professional's experience were not explored and may be the subject of future studies.

## CONCLUSIONS

In the present study, difficulties with the application of the criteria for brain death were identified in a significant portion of the sample. However, the following factors were significantly associated with greater ease in applying the brain death criteria: age between 31 and 41 years; more years of training; greater confidence in explaining the concept to family members; recognition of the need for complementary tests; and a higher number of brain death protocols initiated. These factors corroborate the criteria defined in the new resolution of the Federal Council of Medicine, resolution 2,173 from 2017. The data presented reinforce the relevance of continuous training for professionals on the topic, especially at this time of change of brain death diagnostic standards, seeking greater awareness and training of those involved and, potentially, increasing the number of organ donors.

## Annex 1

**Annex 1:** Questionnaire

QUESTIONNAIRE: (correct alternatives in bold)

**SECTION I:**
**1.** Age: _____

**2.** Sex: ( ) Female ( ) Male

**3.** Year of training: ________

**4.** Specialty: ( ) clinical ( ) surgical

**5.** Did you have prior knowledge of this study?
1. Yes 2. No

**6.** Work predominantly in the ICU:
1. Yes 2. No
- If yes, for how long? _____ years

**7.** What is your main or most important activity?
1. On duty 2. On daily shift (5 times/week)
3. Resident 4. Teacher/supervisor

**SECTION II:**
**1.** Do you have any difficulty following the criteria for brain death?
1. Yes 2. No

**2.** How many times have you initiated the brain death protocol? ________

**3.** How do you rate your confidence to explain to a patient's family what brain death is?
(no confidence) (great confidence)
1 2 3 4 5

**4.** Is the hospital structure adequate for establishing the diagnosis of brain death?
1. Yes 2. No

**5.** Do you think that you received sufficient information about this topic during your medical program?
1. Yes 2. No

**6.** Would you provide a declaration of brain death based only on clinical examination?
1. Yes **2. No**

**7.** What brain functions should be absent for a person to be declared brain dead?
1. Irreversible loss of cerebral cortical function.
**2. Irreversible loss of all cortical and brainstem function.**
3. Variable according to the law.
4. I do not know.

**8.** Is there a legal need for complementary tests to establish the diagnosis of brain death?
**1. Yes** 2. No
If yes, which of these tests can be used for diagnosis?
**1. Arteriography.**
**2. Electroencephalogram (EEG).**
3. Cerebrospinal fluid examination.
**4. Transcranial Doppler.**
5. Simple brain computed tomography.

**9.** A 5-year-old girl is found at the bottom of a swimming pool. She initially presents with apnea and no pulse. She undergoes exhaustive resuscitation measures. After 1 week in the ICU, she does not present corneal, cough or gag reflexes. She does not respond to pain stimuli. There is no nystagmus in response to caloric tests. Apnea test is positive. The results do not change in the next 2 days. Based on this information, your approach would be:
1. After informing the parents and obtaining their consent, remove her from life support.
**2. Request a cerebral blood flow evaluation.**
3. Remove her from life support without the knowledge of the parents.
4. Declare her brain dead.

**10.** An adult patient has a first clinical examination compatible with brain death at 12:00 pm on August 10. The second clinical examination is performed at 6:00 pm* on the same day and remains unchanged. The patient is kept on vital support until suffering cardiac arrest at 8:00 pm on August 11. What is the time of death that should appear on the death certificate?
1. Time of the first clinical examination (12:00 pm on August 10).
2. Time of the second clinical examination (6:00 pm on August 10).
**3. Time of cardiac arrest (8:00 pm on August 11).**

**11.** If the previous patient was an organ donor, what would be the time of death?
1. Time of first clinical examination or initiation the protocol (12:00 pm on August 10).
**2. Time of second clinical examination or of the end of the protocol (6:00 pm on August 10).**
3. Time of removal of organs.
